# Advancing Immune and Cell-Based Therapies Through Imaging

**DOI:** 10.1007/s11307-017-1069-7

**Published:** 2017-03-07

**Authors:** Vladimir Ponomarev

**Affiliations:** Department of Radiology, Molecular Pharmacology and Chemistry Program, Sloan Kettering Institute, Memorial Sloan Kettering Cancer Center, 1275 York Ave Z-2063, Box 501, New York, NY 10065 USA

**Keywords:** Cell-based therapy, Imaging, Immunotherapy

## Abstract

Immunotherapies include various approaches, ranging from stimulating effector mechanisms to counteracting inhibitory and suppressive mechanisms, and creating a forum for discussing the most effective means of advancing these therapies through imaging is the focus of the newly formed Imaging in Cellular and Immune Therapies (ICIT) interest group within the World Molecular Imaging Society. Efforts are being made in the identification and validation of predictive biomarkers for a number of immunotherapies. Without predictive biomarkers, a considerable number of patients may receive treatments that have no chance of offering a benefit. This will reflect poorly on the field of immunotherapy and will yield false hopes in patients while at the same time contributing to significant cost to the healthcare system. This review summarizes the main strategies in cancer immune and cell-based therapies and discusses recent advances in imaging strategies aimed to improve cancer immunotherapy outcomes.

## Introduction

Recently, immuno-based therapies have been found to provide lasting and curative benefits to patients who have previously had very few treatment options available to them. These immunotherapeutic approaches thereby have the potential to revolutionize cancer therapy and become an important part of comprehensive therapeutic approaches for treating many diseases including cancer. These therapies build on the primary function of the immune system to rid the body of threats, both “foreign”, such as bacterial and viral pathogens and “domestic”, such as cancer cells. Over the past several decades of cancer immunology research, there has been evidence that tumor cells are recognized by the native or genetically engineered adoptive immunity, but also that tumors can modify these responses and escape immune recognition [1]. Immune modulation in cancer treatment, therefore, refers to a range of approaches aimed at harnessing and enhancing the patient’s immune system and overcoming immune escape, to achieve tumor control, stabilization, and potential eradication of disease. This has led to the development of therapies that permit specific tumor destruction with minimal toxicity to normal tissue [2–6]. Such strategies also have the potential to prevent tumor recurrence because of the immune systems long-term memory. Within the World Molecular Imaging Society, we have constituted an interest group comprised of members of the society who share the common interest of fostering the use of imaging to advance and understand cellular and immune-based therapies. This Interest Group is called Imaging in Cellular and Immune Therapies (ICIT), and we present here a brief summary of the field and our efforts in the society to support developments in this important area of investigation.

## Current Advances in Immune and Cell Therapies

The main categories of immunotherapies being developed include monoclonal antibodies, recombinant cytokines, cancer-specific, and other types of vaccines and adoptive immune cell transfer [3, 7, 8]. Several immunotherapies have been approved by the Food and Drug Administration (FDA), and others are being evaluated in pre-clinical and clinical trials. The field of immunotherapy for cancer has received a significant boost by the approval of several immunotherapies. These include immunostimulatory cytokines for the treatment of cancer, viral hepatitis and osteopetrosis (*e.g.*, IL-2 [9], interferons [10, 11]). The autologous cellular vaccine, sipuleucel-T, has also recently been approved for the treatment of prostate cancer [12]. Perhaps the immunotherapies that have received the most attention are the immune checkpoint inhibitors that overcome immune escape. These target the anti-cytotoxic T lymphocyte-associated protein 4 (anti-CTLA-4) and the anti-programmed cell death protein/protein ligand 1 (anti-PD-1/PD-L1) with antibodies and are being used for the treatment of melanoma, lung cancer, renal cell carcinoma and head and neck tumors [13–16]. Other forms of immunotherapy including administration of *ex vivo* expanded tumor infiltrating lymphocytes (TILs), transgenic endogenous T cell receptor (TCR)- or chimeric antigen receptor (CAR)-grafted T cells have been successfully tested in clinical trials in patients with melanoma, B cell malignancies, mesothelioma, ovarian and prostate cancers and will likely get FDA approval in the near future [17–23]. This emerging collection of immune-based therapeutic approaches hold great promise for the treatment of cancer, but are also be adopted for many non-malignant conditions such as autoimmunity, infectious diseases, and immunodeficiencies [24–26].

At the moment, immunotherapy with checkpoint inhibitors provide a foundation for many of the combinatorial strategies [27] as it allows for enhancing an immune response against multiple cancers including tumors expressing weak antigens [28]. However, one of the main obstacles to the development of a successful immunotherapeutic approach is in identification and *in vivo* assessment of the most suitable antigen(s) to use. Molecular imaging reagents that target specific cancer antigens can help selecting patients that will likely respond to antigen-directed immunotherapies and will allow for early response assessment and prediction of treatment outcome by visualizing antigen distribution preceding tumor targeting with radioimmunotherapy, drug-immunoconjugates, or antigen-specific T lymphocytes [29–33].

## Imaging as an Outcome Measure

A vital component of any cancer treatment is the objective assessment and monitoring of tumor response to anticancer therapy using imaging and specific response evaluation criteria. The response evaluation criteria in solid tumors (RECIST) was proposed in 2000, and this criterion is anatomic in nature and specifies the number of disease sites and their dynamics as the imaging metric for determining response (*e.g.*, complete response (CR), partial response (PR), stable disease (SD), and progressive disease (PD)) [34]. However, clinical experience suggests that RECIST may be inadequate to cover the spectrum of imaging responses to immunotherapy since the determinants of increase in tumor size and/or the appearance of new lesions that often suggest treatment failure, might not be entirely applicable here. As observed in multiple studies, immunotherapy often demonstrates initial anatomical imaging changes that would be classified as disease progression, followed by radiological and clinical response suggesting therapeutic response [35, 36].

As immunotherapies continue to be developed and undergo testing in clinical trials, consideration needs to be given to the unique appearance of images that predict tumor responses. Such studies have found four distinct response patterns on imaging, all of which were associated with favorable survival: (a) shrinkage of baseline lesions without new lesions, which is consistent with RECIST; (b) durable SD followed by a slow, steady decline in total tumor burden in some patients; (c) response after initial increase in total tumor burden; and (d) response in the presence of new lesions [37–39]. These updated immune-related response criteria (IrRC) are defined as a way to incorporate imaging patterns observed with immunotherapy into the response assessment criteria including metabolic response to treatment assessed by 2-deoxy-2-[^18^F]fluoro-D-glucose ([^18^F]FDG) and positron emission tomography (PET) [40]. One of the aims of the ICIT Interest Group is to verify and validate these measures over a range of immune-based therapies, and to continue to develop new measures as new tools and technologies are developed for both treating and imaging cancer.

## Imaging Biomarkers

One of the challenges of immunotherapy is that accurate and reproducible biomarkers that would allow treating physicians to select the patients most likely to respond have yet to be identified, vetted, and deployed. The potential of molecular imaging to detect these biomarkers and better stratify the patients for targeted therapies and interpreting their response to this novel treatment option cannot be overestimated. Currently, there is no single robust biomarker to identify the patients who will most likely benefit from these treatments, and as we advance precision medicine, it is likely that panels of biomarkers will be required for guiding therapy and assessing outcome. For example, PD-L1 expression levels have been suggested as a positive prognostic biomarker for patients undergoing immune checkpoint blockade therapy [41]. Following pathology-derived data, a number of PD-L1-specific radiotracers were developed that allowed for non-invasive assessment of tumor PD-L1 status in murine models, with the intent of predicting the efficacy of PD-1 checkpoint blockade [42–45]. However, studies have demonstrated the lack of clinical benefit in some patients treated with anti-PD-1 antibodies despite positive PD-L1 status of their tumors [46, 47]. Therefore, the levels of PD-L1 within the tumor microenvironment cannot, at present, be considered an optimal biomarker for patient selection until we can better reveal and understand the basic biology and mechanisms of action of check-point blockade.

There are certain challenges with [^18^F]FDG and [^18^F]FLT PET avidity as biomarkers in assessment of the efficacy of immunotherapy that include the difficulty of discriminating between viable tumor and the infectious or inflammatory processes associated with novel therapies [48, 49]. A number of new radiopharmaceuticals have been proposed and evaluated in pre-clinical and clinical studies aiming to distinguish rapidly proliferating immune cells invading the tumor or involved in any type of immune response. Several ^18^F–labeled nucleoside analogs, including 1-(2′-Deoxy-2′-[^18^F]fluoroarabinofuranosylcytosine (aka [^18^F]FAC) [50, 51], 2-chloro-2′-deoxy-2′-[(18)F]fluoro-9-β-d-arabinofuranosyl-adenine (aka [^18^F]CFA) [52, 53], and 2′-deoxy-2′-[^18^F]fluoro-9-β-D-arabinofuranosylguanine (aka [^18^F]AraG) [54] that are specific for key enzymes involved in T lymphocyte and other immune cell activation and proliferation, have been used for detecting location of activated T cells, monitoring transplant rejection, and graft–*versus*–host–disease and diagnosis and staging of auto–immune disorders. These new radiotracers can be early predictors of response and adverse reactions to immunotherapy measuring functional status of immune cells involved.

**Fig. 1:**
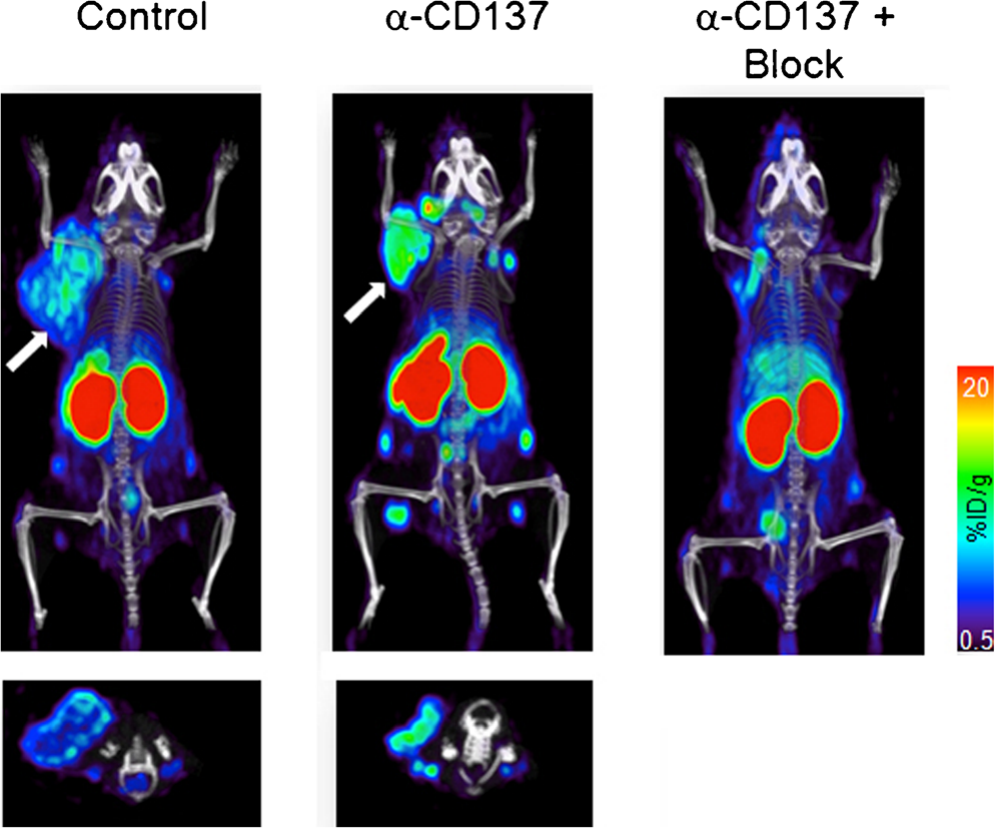
Anti-CD8 immuno-PET in subcutaneous colorectal immunotherapy model. CT26 murine colon cancer-grafted mice were treated with CD137-agonistic Abs: CD137-treated, CD137-treated/CD8-blocked mice, and control mice (no anti-CD137 therapy) were injected with 89Zr-malDFO-169 cDb and immuno-PET images were acquired at 22 h postinjection. Note the presence of increased CD8+ tumor-infiltrating lymphocytes within the tumor in CD137-treated animal (*white arrow*, adapted from [56]).

Another imaging approach that can directly visualize immune cells behavior is immuno-PET. In this approach, antibodies or antibody fragments are used to direct PET radionuclides to immune cells for detection, localization and typing. Immuno-PET has the potential to detect T cell subsets within tumors or lymphoid tissues and noninvasively monitor distribution and temporal dynamics of CD8 cytotoxic and CD4 helper T lymphocytes that participate in the inflammation and cytotoxic attack within tumors. Anti-CD8 full size antibodies and mini-bodies labeled with Cu-64, Zr-89, F-18, or I-124 showed excellent PET imaging and target specificity and importantly did not deplete CD8 T cells due to the absence of Fc function (Fig. 1) [55, 56]. Other targets include markers of immunostimulatory dendritic cells (MHC II, IDO1), chemokine ligands, and receptors (CXCR3,-4, CXCL9,-12 CCR5) as well as immunosuppressive neutrophils and macrophages (CD11b, CD47), which may downregulate T cell responses within tumors [57–61]. Examples of immuno-imaging targets and agents are summarized in Table 1.

**Table 1:** Immuno-imaging targets and agents

Immune cell compartment/function	Molecular target	Imaging agent	Modality	Stage of development
Tumor/Tumor immunosuppressive microenvironment	PD-L1	[^89^Zr]/[^64^Cu]Anti-PD-L1-Ab/peptide [43−45] ([^89^Zr]MPDL3280A) [NCT02453984]	PET	Clinical
Immune cell proliferation	Deoxycytidine kinase	L-[^18^F]FAC, −CFA [51, 53]	PET	Clinical
T lymphocyte activation	Deoxyguanosine kinase	[^18^F]AraG [54]	PET	Clinical
Cytotoxic T lymphocytes	CD8	[^89^Zr]/[^64^Cu]Anti-CD8-Ab [55, 56] ([^89^Zr]IAB22M2C)	PET	Clinical
Macrophages	N/A	Ferumoxytol [62]	MRI	Clinical
T lymphocyte inhibition	CTLA-4	[^64^Cu]Anti-CTLA-4-Ab [63]	PET	Pre-clinical
T lymphocyte inhibition	PD-1	[^64^Cu]Anti-PD-1-Ab [45]	PET	Pre-clinical
Macrophages (TAMs)	CD47	[^89^Zr]Anti-CD47-Ab [58]	PET	Pre-clinical
Neutrophils	CD11b/MHC-II	[^18^F]/[^64^Cu]Anti-CD11b/MHC-II-Ab [57]	PET	Pre-clinical
Transgeneic T cells	Transgeneic TCR	[^89^Zr]/[^64^Cu]Anti–TCR-Ab [64, 65]	PET	Pre-clinical
T lymphocyte trafficking	CXCR4	[^64^Cu]AMD3100 [60]	PET	Pre-clinical
Macrophages (TAMs)	B7-H3	MicrobubblesAnti-B7-H3 [66]	Ultrasound	Pre-clinical

*N/A* not available

## Imaging of Immune Cells

Recent advances in the field of adoptive immunotherapy require the ability to monitor the trafficking, targeting, and activation/proliferation of the administered cells. The application of labeling molecules and genetic reporter systems (gene/probe combinations) together with non-invasive imaging modalities, such as PET, SPECT, and MRI, has shown the potential for monitoring T cells in clinical settings [67–69]. In-111, in particular, found a wide clinical application in oncology as an imaging agent for monitoring immunotherapy with tumor-infiltrating lymphocytes and granulocytes administration [70, 71]. However, imaging approaches that require *ex vivo* cell labeling encompass a number of limitations such as radiotoxicity and limited period of monitoring as a result of cell division, biological clearance and radiolabel decay [72, 73].

**Fig. 2:**
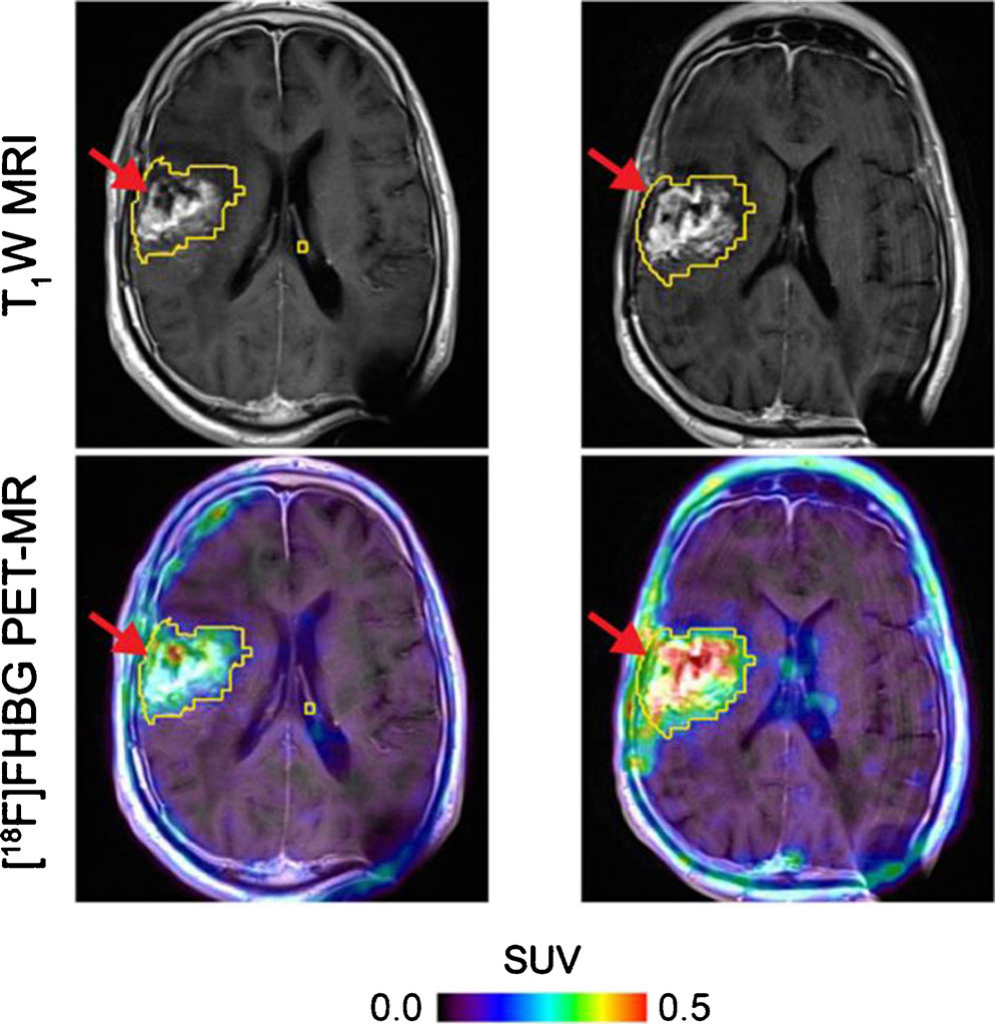
Tumor-directed CAR-T cell PET imaging with [^18^F]FHBG. [^18^F]FHBG PET imaging was performed in a patient with a recurrent right frontoparietal glioblastoma **a** before and **b** 1 week after tumor-specific CAR-T cell infusions. Allogeneic CAR-T cells and IL-2 were injected intratumorally (*red arrows*). Tumor recurrence was monitored by T_1_-weighted (T_1_W) MRI (top panels). [^18^F]FHBG PET images were fused with MR images (*bottom panels*), and three-dimensional (3D) volumes of interest were drawn using a 50 % [^18^F]FHBG SUV_max_threshold, outlined in *yellow* (adapted from [80]).

Stable genetic labeling of adoptively transferred cells with reporter genes (genes encoding easily detectable proteins not normally expressed by the cells) has been used to circumvent the temporal limitations of *ex vivo* radiolabeling. Several reporter gene/reporter probe combinations have been used in the majority of the seminal studies on imaging immune cell trafficking including *ex vivo* expanded cytotoxic lymphocytes (CTLs) and CAR-grafted Tcells by optical and nuclear techniques (*e.g.*, luciferases, fluorescent proteins, HSV1tk, respectively [67, 68, 74–79]) including imaging in humans (Fig. 2) [80]. In addition, reporter gene imaging allows for visualization of T lymphocyte functional status following T cell receptor engagement by using inducible reporter systems sensitive to T cell activation [81]. This circumstance makes this imaging approach clinically valuable, as it allows for monitoring functional status of adoptively transferred immune cells in cancer patients as well as in bone marrow and stem cell recipients. The major impediment to the translation of viral- and bacterial-derived reporter gene imaging approaches into clinical practice is the immunogenicity of these non-human derived reporter proteins. However, there has been a recent focus on human reporter systems to avoid the potential risk of generating an immunological reaction to xenogeneic (non-human) reporter proteins thus allowing for long-term repetitive visualization of adoptively transferred immune cells. These include human-derived sodium iodide symporter (hNIS), norepinephrine transporter (hNET), somatostatin receptor 2 (hSSTR2), truncated, and mutated mitochondrial thymidine kinase type 2 (h∆TK2, h∆TK2DM) and deoxycytidine kinase (hdCKDM, hdCK3M), ferritin reporter, and transferrin receptor [82–89]. Some reporter genes may have the added benefit of suicide gene potential. This provides a mechanism for elimination of rogue reporter gene-expressing immune cells with clinically approved chemo- and radiotherapeutics (*e.g.*, HSV1tk/ganciclovir, hdCKDM/gemcitabine, hNIS/I-131, hNET/[^131^I]MIBG [90–93]).

Principles of immuno-PET can be applied to imaging adoptively transferred cells by administrating anti-antigen-specific transgenic TCR radiolabeled antibodies. Antigen-specific TCR transgenic T cells were successfully visualized with PET using ^89^Zr-89 labeled anti–TCRmu-F(ab')_2_ fragment [64] and [^64^Cu]DOTA–modified cOVA-TCR–specific mAbs [65].

The potential of imaging for quantifying cell signals in a region of anatomical interest (ROI) provides a unique opportunity to estimate the absolute number of injected labeled cells at the target site. Several studies determined the correlation of PET signal to cell number and characterized the cellular limit of detection for PET imaging using human and mouse T cells transduced with different human and non-human reporters with a limit of detection below 10^5^ cells in a region of interest of 0.1 ml volume [85, 87, 94]. This level of sensitivity enables effective assessment of cell localization at target sites and assessment of off target homing *in vivo* and will be useful in guiding development of novel immune therapies.

## Conclusion

As immunotherapies continue to be developed and undergo testing in clinical trials, consideration needs to be given to the differences observed in response following immunotherapy, and as a community, we need to standardize these measures and ensure uniformity among studies. While optimal combinations of treatment schemas still need to be determined, significant efforts have to be made in the identification and validation of predictive biomarkers that can be used alone or in combination in imaging, but also in conjunction with blood and tissue markers *ex vivo*. It is imperative that we as a community of imaging scientists interested in immunotherapy be proactive in advancing this field.
